# Nutrition rehabilitation of undernourished children utilizing Spiruline and Misola

**DOI:** 10.1186/1475-2891-5-3

**Published:** 2006-01-23

**Authors:** Jacques Simpore, Fatoumata Kabore, Frederic Zongo, Deleli Dansou, Augustin Bere, Salvatore Pignatelli, Daniela M Biondi, Giuseppe Ruberto, Salvatore Musumeci

**Affiliations:** 1 Unit of Formation and of Research in Sciences of Life and of the Earth, University of Ouagadougou, Burkina Faso, Africa; 2 Medical Centre St Camille (CMSC), Ouagadougou, Burkina Faso, Africa; 3 Institute of Biomolecular Chemistry, National Research Council (CNR), Via del Santuario 110, Valverde 95028, Italy; 4 Department of Pharmacology, Gynaecology and Obstetrics, Paediatrics, University of Sassari, Viale san Pietro 43b, Sassari 07100, Italy; 5 Institute of Population Genetics, National Research Council (CNR), S.P. 95, Km 8.400 Loc Tramariglio, Alghero 07041, Italy

## Abstract

**Background:**

Malnutrition constitutes a public health problem throughout the world and particularly in developing countries.

**Aims:**

The objective of the study is to assess the impact of an elementary integrator composed of Spiruline (*Spirulina platensis*) and Misola (millet, soja, peanut) produced at the Centre Medical St Camille (CMSC) of Ouagadougou, Burkina Faso, on the nutritional status of undernourished children.

**Materials and methods:**

550 undernourished children of less than 5 years old were enrolled in this study, 455 showed severe marasma, 57 marasma of medium severity and 38 kwashiorkor plus marasma. We divided the children randomly into four groups: 170 were given Misola (731 ± 7 kcal/day), 170 were given Spiruline plus traditional meals (748 ± 6 kcal/day), 170 were given Spiruline plus Misola (767 ± 5 kcal/day). Forty children received only traditional meals (722 ± 8 kcal/day) and functioned as the control group. The duration of this study was eight weeks.

**Results and Discussion:**

Anthropometrics and haematological parameters allowed us to appreciate both the nutritional and biological evolution of these children. The rehabilitation with Spiruline plus Misola (this association gave an energy intake of 767 ± 5 kcal/day with a protein assumption of 33.3 ± 1.2 g a day), both greater than Misola or Spiruline alone, seems to correct weight loss more quickly.

**Conclusion:**

Our results indicate that Misola, Spiruline plus traditional meals or Spiruline plus Misola are all a good food supplement for undernourished children, but the rehabilitation by Spiruline plus Misola seems synergically favour the nutrition rehabilitation better than the simple addition of protein and energy intake.

## Introduction

Malnutrition constitutes a public health problem throughout the world and particularly in developing countries [1]. In Africa, more than 30% of the deaths of less than five year old children result directly or indirectly from malnutrition [2]. Since 1999, Burkina Faso has been confronted by protein-energy malnutrition with 13 % of the infant population affected by emaciation, 29% by growth retardation and 30% by insufficient weight [3]. The consequences of the protein-energy malnutrition in Burkina Faso are several, but especially severe forms of marasma, kwashiorkor and marasmic kwashiorkor are manifested [3]. Today it is recognized that this form of malnutrition is coupled with deficiencies in vitamins and minerals [4,5]. It inexorably creates a spiral between malnutrition and infectious pathologies, which are often associated with chronic diarrhoea and compound the prognosis of these children [6]. In the Nutrition Education and Rehabilitation Centre (CREN) of Ouagadougou, Burkina Faso, Misola or Spiruline or both together have been used since 2000 to improve the nutritional status of undernourished children. The choice of these two elementary integrators was prompted by the biochemical composition of both. Misola, a local flour traditionally produced at the CREN of the Centre Medical St. Camille (CMSC) of Ouagadougou which contains millet, soja, peanut following the original formula proposed by the Association Burkinabe des Unites Misola and powder of Spiruline, a cyanobacterium which grows easily under the climate of Burkina Faso, also produced at the CMSC, were recently introduced in the treatment of undernourished children for its biological activities [7]. Spiruline was utilized for its elevated content of aminoacids, iron and carotenoids. Spiruline used in this study was also analysed for its chemical composition since its lipid composition is influenced by the environmental growing conditions.

## Subjects and methods

**Figure 1:**
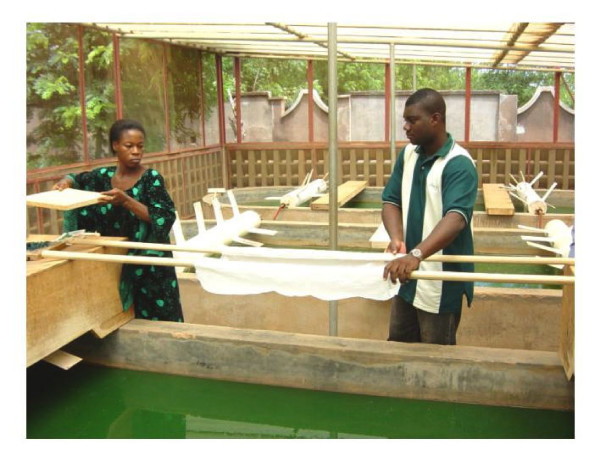
Particular of bacins for Spiruline cultivation.

This research was conducted at the CMSC of Ouagadougou during 2002–2003. The centre was created in 1974 by the St Camille religious order and comprises a maternity centre, a health centre, an analysis laboratory for biological and biochemical examination, a centre for neonatal pathology, a greenhouse for growing the Spiruline (Figure 1) and a Nutrition Education and Rehabilitation Centre (CREN). The CREN follows on average 700 children per year.

### Study protocol

Infants and children aged <5 years were enrolled using the CONSORT criteria [8]. Each child was admitted to the protocol study and given a progressive number and each was selected randomly with a casual number generator program. Dehydration resulting in shock requires rapid transfer to the hospital for intensive therapy (exclusion criteria). Discontinuation criteria were abandonment, death and the interruption of treatment at the CMSC during the study.

### Study patients

At the beginning of this study, undernourished children were anoretic and many of them had diarrhoea, which was treated with nose-gastric (NG) re-hydration according to the CMSC protocol [6]. They were taken off NG feeding before being selected for this study, since this condition had sufficiently improved to allow moving on to oral feeding. At the end, 550 children were enrolled randomly in three rehabilitation re-hydration protocols: A) 170 of them received an alimentation with only Misola, B) 170 were treated by supplementing Spiruline to traditional meals (millet, vegetable, fruit), C) 170 received Spiruline plus Misola. A control group of 40 undernourished children from the same age range was chosen randomly between children whose mothers did not accept the protocol study, so they were fed only with traditional meals. The vitamin and mineral deficiencies were corrected only at the end of study.

### Participation criteria

All studied children were undernourished according to the z-score criteria, recommended by the WHO and the United Nations Children's Fund (UNICEF), and their median age was 15.29 months (6–60 months). The ages were confirmed by their birth notebooks. The Ethical Committee of CMSC gave permission for the study and all parents were informed of its aims. They gave written consent for the participation of their children in the study.

### Anthropometric parameters

The weight of the children was taken once a week starting from the day of admission to the CREN with a 10 grams sensitivity balance. The recumbent length is measured by resting the child on his back; children over 2 years are measured in an upright position.

The nutritional status, evaluated by brachial parameters was compared to the classification of Jelliffe [9], considering that it varies little for the children of less than four years.

HAZ (Height for age z-score), WHZ (Weight for height z-score) and WAZ (Weight for age z-score) parameters were calculated according to the references of the National Centre for Health Statistics (NCHS) [10].

### Evaluation of results

The evaluation of the nutritional status of the children has been made according to the nutritional indices. The weight for age index expressed in z-score (WAZ) or weight insufficiency indicates a global malnutrition affecting both the linear growth and the weight increment. The height for age index expressed in z-score (HAZ) or growth delay is an index indicating chronic malnutrition provoked by an extended reduction of food consumption and by repeated pathologic episodes. Emaciation or weight loss expressed by the weight for height index (WHZ) indicates a slightly less malnutrition status or weight deficit due to a decrease or slowdown of regular growth. These tests were performed to obtain significant changes within the treatment groups in order to detect whether Spiruline or Misola are a useful supplement for rehabilitation.

### Plant material

Spiruline was cultivated in Burkina Faso, in artificial ponds and dried at room temperature. The material was stored in the dark at 4°C to prevent photodegradation.

### Preparation and administration of Spiruline and Misola

The mothers of the undernourished children to receive Spiruline or Spiruline plus Misola were given weekly rations of Spiruline in a sachet. Every day, they had to mix 5 g of Spiruline with the traditional meal of their children composed of 50 grams of millet flour in a graduated container. Other mothers added 5 g of Spiruline to 50 grams of Misola flour. These integrations were made at least twice a day. The Misola, a kind of bouillon, is a mixture of millet (60%), soya (20%), peanut kernel (10%), sugar (9%) and salt (1%). The preparation of the Misola or millet was carried out according to traditional customs, namely 50 grams of Misola or millet and 200 ml of water were mixed and boiled over a low fire, mixing for 2–3 minutes. Each mother fed own child 4 times a day (6:30; 10:30; 14:30 and 18:30),  two with the integrator (Spiruline) and two without. The mothers rapidly learned to prepare the mixtures and feed their children inside the CREN. A simple twenty-four hour diet recall was used in a printed double A4 sheet of paper, with the aim to obtain reliable data for energy and protein intake. It asked for a record of all foods, taken over the day. A written example was included. Every morning the sheet was collected, the children were weighed and the exact quantity of food ingested clearly annoted in a register. After this preliminary phase they continued to administer the mixture at home. Each day they accompanied their children to the CREN to monitor weight and other anthropometric parameters and delivered the 24 hours diet recall sheet to CREN. At the end of the sheet, a number of additional questions were asked, such as the appetite, liveliness, humour.

### Chemical studies

The fatty matter content was determined by the Soxhlet method of the extraction. The ≪total protein≫ or ≪total nitrogen≫ fraction was measured by the Kjeldahl method. The content of glucides was determined by a colorimetric dosage or orcinol method. The lipid composition was evaluated by the analysis of fatty acid methyl esters (FAME).

### Fatty acids quantification and identification

The Spiruline was ground and extracted three times with hexane. The mixture of fatty acid methyl esters has been extracted with hexane and analyzed by Hewlett Packard gas-chromatograph, Model 5890, equipped with a flame ionization detector (FID) and coupled to an electronic integrator. The components were identified by using standard fatty acid methyl esters and quantified by using methyl nonadecanoate (19:0) as an internal standard.

### Statistic analysis

A power analysis was performed prior to the initiation of the study and the number of studied children was homogeneously distributed. The study reached the minimal number of observations to discuss a statistical difference. The data were treated with Excel (Office, Microsoft) software, Epi-Info software V. 6 for the anthropometric data and SPSS-10 for biological data, according to the opportunities of calculations and of analysis. The difference between mean values before and after eight weeks of treatment were calculated by Student T test. P < 0.05 was considered significant.

## Results

### Nutritional rehabilitation

All randomly chosen children completed the eight weeks of treatment. Table 1 shows the anthropometric parameters of the children at the beginning of our study. The baseline anthropometric status was equivalent among the groups, with the exception of HAZ for group C (- 2.64). Moreover, according to HIV serology, no significant differences are observed in these parameters: HAZ, WHZ, WAZ and the BP.

**Table 1:** Anthropometric parameters of the children subjected to the study.^a^

	**A **170 Children with Misola 200 g/day	**B **170 Children with Spiruline 10 g/day plus traditional meals 200 g/day	**C **170 Children with Spiruline 10 g/day plus Misola 200 g/day	**D **40 children with traditional meals 200 g/day	**Variance Analysis**
Age (months)	15.39 ± 8.3	14.96 ± 5.9	13.86 ± 8.5	15.19 ± 4.35	P = NS
Height (cm)	67.00 ± 8.3	69.84 ± 5.8	69.06 ± 8.5	68.24 ± 4.5	P < 0.01
B.P.	11.17 ± 1.2	10.40 ± 1.0	11.20 ± 1.2	10.37 ± 1.0	P < 0.001
Weight (kg)	6.12 ± 1.4	5.98 ± 1.1	5.99 ± 1.5	6.10 ± 1.2	P = NS
HAZ	-3.93 ± 5.3	-2.64 ± 2.1	-3.35 ± 5.3	-3.23 ± 1.5	P = 0.057
WHZ	-1.73 ± 2.5	-2.88 ± 0.9	-3.05 ± 0.75	-2.32 ± 1.02	P < 0.001
WAZ	-4.01 ± 1.0	-3.88 ± 1.0	-4.38 ± 0.9	-3.99 ± 0.9	P < 0.001
Energy intake (kcal/day)	731 ± 7	748 ± 6	767 ± 5	722 ± 8	P < 0.001
Protein (g/day)	27. 1 ± 1.7	27.8 ± 1.6	33.3 ± 1.2	22.1 ± 1.3	P < 0.001

^a^HAZ = Height for age z-score; WHZ = Weight for height z-score; WAZ = Weight for age z-score, B.P = Brachial Perimeter. NS = not significant

Males were heavier than females with respective significant differences: p < 0.0001 (Table 2).

**Table 2:** Median anthropometric parameters of the children according to sex at the beginning of the study.

	286 Female		264 Males		All children (550)	
Parameters	Mean	Variance	Mean	Variance	Mean	Variance

Age (months)	15.64	8.08	15.01	6.87	15.30	7.41
Weight (Kg)	5.82	1.17	6.28*	1.36	6.07	1.29
Height (cm)	68.07	6.73	68.43	7.48	68.27	7.13
P.B.	10.75	1.13	10.99	1.25	10.88	1.20

Student T test * P = 0.0001

The nutritional pre/post changes improved in all children, but more significantly in the group that received Misola plus Spiruline. These changes among treatment groups are reported in Table 3. This improvement corresponds to an increment of weight which was on average 20 g a day in the Misola group, 25 g a day in the Spiruline plus traditional meals group, 34 g a day in the Misola plus Spiruline group and 15 g a day in the control group. These pre/post differences within groups were statistically significant considering the differences in the nutritional status changes across the groups, but this difference was less significant in the control group.

**Table 3:** Nutritional status at the beginning [1] and end of the study [2].^a^

	A 170 Children with Misola 200 g/day	B 170 Children with Spiruline 10 g/day plus traditional meals 200 g/day	C 170 Children with Spiruline 10 g/day plus Misola 200 g/day	D 40 Children with traditional meals 200 g/day
WHZ1 1 → 2	-1.73 ± 2.51 P = 0.035*	-2.88 ± 0.95 P < 0.001	-3,05 ± 0.75 P < 0.001*	-2.42 ± 1.02 P = 0.065*
WHZ2	-1.14 ± 2.64	-1.80 ± 1.53	-1,18 ± 1.63	-2.00 ± 0.99
WHZ2/WHZ1+WHZ2	34.14 %	37.50 %	62.90 %	17.35 %
WAZ1 1 → 2	-4.01 ± 0.98 P < 0.001**	-3.88 ± 0.90 P < 0.001**	-4,38 ± 0.91 P < 0.001**	-3.99 ± 0.9 P = 0.013**
WAZ2	-2.95 ± 1.12	-3.10 ± 1.14	-2,71 ± 1.17	-3.45 ± 1.0
WAZ2/WAZ1+WAZ2	26 %	20 %	38 %	14 %

^a^WHZ1 = Weight for height z-score at beginning of the study; WHZ2 = Weight for height z-score at the end of the study; WAZ1 = Weight for age z-score at the beginning of study; WAZ2 = Weight for age z-score at the end of the study. Student T test *WHZ1→WHZ2; **WAZ1→WAZ2 ;

At the end of the eight weeks of the treatment, nutritional status normalized for the majority of children, with the WHZ parameter decreasing from -2.26 to -0.93. The index weight for age WAZ at the end of our study allowed to confirm that severe malnutrition was corrected by this protocol of treatment, more significantly in the Misola plus Spiruline group. The percentages of WHZ and WAZ are reported in Table 3. The association of Spiruline and Spiruline plus Misola gave a gain of 61% and 38 % respectively. The gain with traditional meals, Misola and Spiruline plus traditional meals was clearly of minor entity.

The compliance to treatment was excellent and no children dropped out. The mothers reported that the children accepted the mixes and rarely had difficulties in feeding their children. They came to weekly appointments, but only the first and the last visit (eight weeks) were considered in the final evaluation.

### Chemical analysis

Misola is an infantile flour composed of millet, soya, peanut kernel, sugar and salt produced in the CREN of the CMSC (Ouagadougou). Table 4 shows the biochemical composition for 100 grams of Misola used at the CSMC and the lipid composition of this mixture where the fatty acid content is represented by palmitic, linoleic, oleic, γ-linolenic, stearic and palmitoleic acids.

**Table 4:** Nutritive composition of 100 grams of Misola used in the CMSC.

Biochemical Composition	Mean Concentration
Lipid	12 %
Protein	16 %
Glucide	61 %
Calories (kcal/100 g)	410

The composition of the cultivated Spiruline of the CMSC is given in Table 5. The values of the composition of the Spiruline from the CMSC of Ouagadougou are within the interval of values of the international firm Green Flamant [11] and its physicochemical elements do not change with time (p > 0.270). The composition of our Spiruline proves the good quality of the Spiruline from the CMSC. The only difference lies at the level of the value of the glucides. The lower glucide content of the analysed Spiruline in our growing conditions was near the one of Sautier and Tremolieres [12] who in 1975 found a value of 12.4% in the laboratory cultivated Spiruline. The quality of the Spiruline in time – in the first three months of storage – did not show significant changes (Table 6). For longer storage periods some significant changes were detected, such as a decrease in protein content and an increase in pH value.

**Table 5:** The Nutrition Composition for 100 grams of cultivated Spiruline from the Center Medical St Camille in comparison to the given values in literature (Green Flamant,. 1998).

	Our results	Green Flamant values
Water content	4.87%	3–7%
Ash	10.38%	7–13%
Vegetal Fiber	7.81%	8–10%
Lipid	6.00%	6–8%
Protein	57.10%	55–70%
Glucide	13.84%	15–25%

**Table 6:** Physicochemical composition of the Spiruline with time.

*Analysed sample*	*T0 (1*^*th *^*day)*	*T1 (1*^*th*^* month)*	*T2 (2*^*nd*^* month)*	*T3 (3*^*th*^* month)*	*T4 (10*^*th*^* month)*
Protein (%)	57,10	56,22	54,69	52,28	49,22
Formic index (ml NaOH)	4,35	4,20	4,47	5,19	4,81
Total sugars (%)	12,77	16,43	19,59	18,16	16,07
Reductive sugars (%)	1,07	2,52	2,17	1,56	1,62
Fat matter (%)	6,00	7,19	6,69	5,92	7,25
Fatty acids (mg NaOH/g)	6,6	6,0	7,5	6,9	10,2
pH	6,53	6,56	6,36	6,78	7,33
Humidity (%)	4,87	4,86	5,01	4,83	4,42
Ash (%)	10,76	12,12	10,19	11,46	14,44
Phycocyanin (%)	9,76	7,46	6,12	7,32	4,46
Energy value (kcal/100 g)	338	360	363	340	331

Student T test for paired data : T0 → T1 : p = 0.273; T0 → T2 : p = 0.310; T0 → T3 : p = 0.763; T0 → T4 : p = 0.625

The lipid composition of the Spiruline grown in Burkina Faso is listed in Table 7. The fatty acid content is represented by palmitic, linoleic, oleic, γ-linolenic, stearic and palmitoleic acids.

**Table 7:** Fatty acid composition of *Spiruline *strain from Burkina Faso.

**Fatty acid**	**Wt % of total fatty acid**
Palmitic acid, 16:0	28.04
Palmitoleic acid, 16:1	2.69
Stearic acid, 18:0	13.44
Oleic acid, 18:1	18.88
Linoleic acid, 18:2	21.87
γ-Linolenic acid, 18:3	15.08

## Discussion

After eight weeks of study, children treated with Misola, Spiruline plus traditional meals and Misola plus Spiruline appeared clinically improved; their weight increased and many of them showed an increase of Hb levels. This improvement was less significant in the control group, who received only traditional meals. The enrolment of this group might seem unethical among these severely malnourished children, but it was organized by choosing a control group randomly between children whose mothers did not accept the protocol study, so they were treated only with traditional meals. In this way, the influence of being unwilling to participate in a study on caloric and nutrient intake (supplement of Spiruline *vs.* traditional meals) becomes negligible.

The association between Misola plus Spiruline achieved greater gains in terms of weight than the Misola or Spiruline alone. This result is clearly due to higher energy intake (767 ± 5 kcal/day) and to greater protein assumption (33.3 ± 1.2 g/day), which synergically favour the nutrition rehabilitation.

The results of this study prompted us to continue the culture of Spiruline in the CMSC of Ouagadougou in order to utilize the biochemical composition and the beneficial action of this cyanobacterium, which may be considered an alimentary integrator for undernourished children. In the context of low intake of proteins, 10 g a day per inhabitant in Africa against 29 g in Latin America and 63 g in the industrialized countries, and the integration of traditional meals with Spiruline and Misola plus Spiruline (57 % of protein), improve the nutritional and micronutrient requirement for undernourished children [13].

This may be due to the iron content of Spiruline supplement [14], which corrects anaemia owing to deficient iron intake.

This mechanism may be due to the high amount in the lipid fraction of ω-6 derivative, namely γ-linolenic acid [15]. The exclusive presence of ω-6 represents a metabolic gain, since desaturase enzyme could be deficient in undernourished children [16].

Growth recovery was slower than weight recovery and this may have been compounded by the diarrhoea, which was present at the beginning of treatment of these children [17]. In fact, in our eight week study, the variations of weight were more significant owing to the liquid content dehydration associated with malnutrition. The percentage of increment in weight with the association of Misola plus Spiruline confirms the suitability for continuing this kind of combination in undernourished children. A previous study made by Branger et al. [18] in Burkina Faso did not show a significant improvement by adding Spiruline to traditional meals and Misola, but, as considered by the same authors, the results they obtained could be due to the quantity of Spiruline, which was half that used in our study (5 g *vs*. 10 g). Moreover, the present study is more conclusive than the one realized in Dakar by Alling *et al*. [19], where weight gain was less, probably also due in this case, to a reduced supplement in Spiruline.

The anthropometric characteristics varied little according to sex (Table 2), but were different according to the nutritional and serologic status. This observation is the same as the one by Kelly *et al*. [20] in undernourished HIV-infected children with persistent diarrhoea. The strong prevalence of kwashiorkor and/or marasma is characteristic of sub-Saharan Africa, where maize and millet are the staple diet. In fact, high intake of linoleic acid in a diet deficient in other polyunsaturated fatty acids and in riboflavin results in high tissue production of prostaglandin E2 in these countries, which in turn causes inhibition of the proliferation and cytokine production of Th1 cells, mediators of cellular immunity [21]. Diet-associated inhibition of the Th1 subset is a major contributor to the high prevalence of these diseases in sub-Saharan areas.

The high percentage of undernourished children in Burkina Faso puts a considerable strain on medical and nutritional resources and organizations, and this study could suggest a preliminary solution with Spiruline plus Misola or Spiruline plus traditional meal to accelerate nutritional rehabilitation.

## Conclusion

This study shows that malnutrition remains a public health problem in Burkina Faso. The consequences of malnutrition represent a global problem, which affects morbidity as well as mortality. Awaiting the enrolment of these undernourished children in rehabilitation protocols, those in charge of public health services and epidemiologists should work in synergy with nutritionists, bacteriologists and virologists in order to combat malnutrition efficiently.

Misola, which has 61 % of glycides with 410 kcal/100 g, has a higher energy content than the Spirulina which has only 13.84 % of glycides with 338 kcal/100 g. Inversely, Spirulina has 57.10 % of protein and the Misola has only 16 %. At the end the high amount of ω-6 lipid component helped an efficient recovery of the precarious immune system of these children. These characteristics confirm the suitability of supplementing Misola with Spirulina (this association gave an energy intake of 767 ± 5 kcal/day with a protein assumption of 33.3 ± 1.2 g a day), both greater than Misola or Spiruline alone. According to the instructions which the mothers received, involvement of the families of the undernourished children and of the whole community is essential to control the great prevalence of malnutrition in African countries.
